# Effects of Drying Methods on the Antioxidant Properties of *Piper betle* Leaves

**DOI:** 10.3390/molecules29081762

**Published:** 2024-04-12

**Authors:** Kivaandra Dayaa Rao Ramarao, Zuliana Razali, Chandran Somasundram, Wijenthiran Kunasekaran, Tan Li Jin

**Affiliations:** 1 Institute of Biological Sciences, Faculty of Science, University of Malaya, Kuala Lumpur 50603, Malaysia; kivaandra@gmail.com (K.D.R.R.); chandran@um.edu.my (C.S.); 2 The Center for Research in Biotechnology for Agriculture (CEBAR), University of Malaya, Kuala Lumpur 50603, Malaysia; 3 Wari Technologies Sdn. Bhd., 2A-2, Galleria Cyberjaya, Jalan Teknokrat 6, Cyber 5, Cyberjaya 63000, Selangor, Malaysia; wijen@wari.tech (W.K.); lijin@wari.tech (T.L.J.)

**Keywords:** *Piper betle*, drying, total polyphenol content, total flavonoid content, DPPH radical scavenging activity, GCMS analysis

## Abstract

*Piper betle* leaf powder is increasingly utilised as a health supplement. In this study, *P. betle* leaves were subjected to four different drying methods: convective air-drying, oven-drying, sun-drying, and no drying, with fresh leaves as control. Their antioxidant properties were then evaluated using colourimetric assays and GC-MS. Results showed that the sun-dried leaves had the highest (*p* < 0.05) total antioxidant capacity (66.23 ± 0.10 mg AAE/g), total polyphenol content (133.93 ± 3.76 mg GAE/g), total flavonoid content (81.25 ± 3.26 mg CE/g) and DPPH radical scavenging activity (56.48 ± 0.11%), and the lowest alkaloid content (45.684 ± 0.265 mg/gm). GC-MS analysis revealed that major constituents of aqueous extracts of fresh and sun-dried *P. betle* leaves were hydrazine 1,2-dimethyl-; ethyl aminomethylformimidate; glycerin; propanoic acid, 2-hydroxy-, methyl ester, (+/−)-; and 1,2-Cyclopentanedione. In conclusion, sun-dried leaves exhibited overall better antioxidant properties, and their aqueous extracts contained biologically active phytoconstituents that have uses in various fields.

## 1. Introduction

*Piper betle* leaves are from the genus Piper of the Piperaceae family (Table 1). There are over 100 varieties of which about 40 are found in India alone [1]. It is known as Betel leaves in English and commonly referred to as ‘sirih’ or ‘sireh’ by the locals in its place of origin, Malaysia. It is cultivated in India, Indonesia, the Philippines, and many other Southeast Asian and East African countries [2]. *P. betle* is an aromatic creeper with smooth, shiny dorsiventral heart-shaped leaves [3,4]. *P. betle* leaves are important for millions in India as their source of income depends on the supply chain of this crop, from production to processing to sales [5].

*P. betle* is a gem in traditional medicine where its uses range from the Ayurvedic field to TCM (traditional Chinese medicine) and even to Western medicine [6]. Its leaves are consumed after meals as a mouth freshener since it has a pleasant smell and as a digestive stimulant [7]. This edible leaf is known to possess medicinal properties such as anti-inflammatory and antioxidant activities [8]. It has seen uses in traditional medicine as a wound-healing agent and to improve digestion [9]. These biological activities are attributed to their high antioxidant activities and compounds [10]. The consumption of antioxidant-rich products like *P. betle* leaves can especially help to neutralise free radicals in our body, thus preventing or delaying the oxidative damage of lipids, proteins, and nucleic acids [11]. Various extractions, like aqueous extractions, were performed on the leaf, and gas chromatography-mass spectrometry (GC-MS) was commonly used to identify the phytochemical constituents [6]. These compounds include alcohols, esters, aldehydes, and alkanes [6,12].

Drying is a process of moisture removal. It has been employed as a method for the drying of leaves for decades [10]. Drying methods involve heat application on a product to remove moisture, and this removal of water can help prevent the decomposition of phytochemicals and microbial contamination in the product [4]. The leaves’ moisture content is usually reduced quickly to prevent enzymic reaction and oxidation, which often damages bioactive compounds in the leaf [10]. There is also a 35 to 75% loss of fresh leaf produce during storage and transport, which causes economic and environmental waste [13]. Therefore, drying is seen as a desirable method for the preservation of bioactive compounds like antioxidants in leaves. A recent study by Thi et al. [14] showed that dried *P. betle* leaves contained higher amounts of antioxidants than fresh leaves. This is because drying helps to concentrate the nutrients in the leaves [15]. Another study by Sahu et al. [16] showed that drying temperatures affect the antioxidant activity of *P. betle* leaves, specifically at high temperatures (80 °C) where polyphenol oxidases could have degraded, causing a reduction in the antioxidant activity. The methanolic extract of dried *P. betle* leaves was shown to contain potent antioxidant compounds like hydroxychavicol, which reduced inflammation by mediating the downregulation of the NF-κB and MAPK pathways [17]. It is also worth noting that these bioactive compounds in the leaves vary based on factors like geographical origin; therefore, it is important to not overgeneralise and continuously monitor their quality as health-promoting foods.

## 2. Results

**Figure 1:**
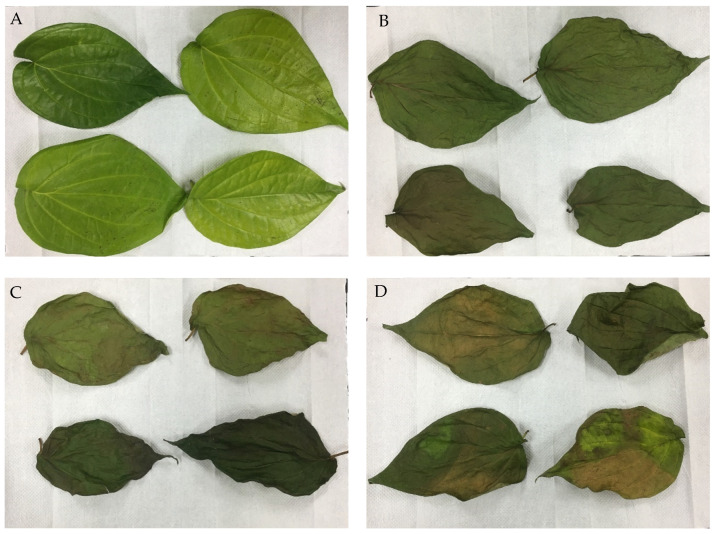
Pictures of *Piper betle* leaves subject to various drying conditions: (**A**) fresh leaves; (**B**) oven-dried leaves; (**C**) convective air-dried leaves; (**D**) sun-dried leaves.

The effects of various drying methods on the antioxidant properties of *P. betle* leaves were studied. The time taken for the betel leaves to dry was 4.5 h, 3 h, and 6 h in the oven, in the convective air-dryer, and under the sun respectively. The dried samples’ percentage weight loss was 81.57% in the oven, 81.90% in the convective air-dryer, and 79.37% under the sun. Figure 1 presents the pictures of the leaves before drying (fresh leaves) and after they were subjected to each drying method, whereas Table 2 depicts the colour parameters as L*, a*, and b* values as measured using the chroma meter.

From Figure 1, it can be seen that the leaves darkened post-drying compared with the control (fresh leaves). This was supported by the data from the chroma meter’s measurement where, as shown in Table 2, the L* values decreased (*p* > 0.05) in all three drying methods compared with fresh leaves, indicating that dried leaves became darker. In addition to that, the a* values were observed to have significantly increased (*p* < 0.05) while b* values significantly decreased (*p* < 0.05) for sun-dried leaves compared with fresh leaves.

**Figure 2:**
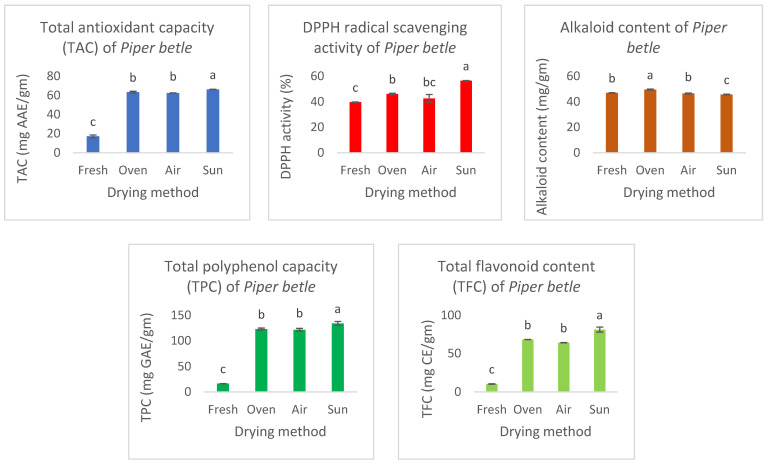
The TAC, DPPH radical scavenging activity, TPC, and TFC of *Piper betle* leaves subject to various drying treatments. Results were expressed as mean ± SD from three experiments (*n* = 3). One-way ANOVA was carried out for each treatment (*p* < 0.05) and showed significance. Post hoc analysis (Tukey HSD test) was used to identify which pair(s) in each column was/were statistically different. The same letter denotes mean values that are not significantly different (*p* > 0.05).

Based on Figure 2, TAC was the highest in sun-dried *P. betle* leaves (66.23 ± 0.10 mg AAE/g), while TAC in the fresh sample was significantly lower (*p* < 0.05) at 17.14 ± 1.44 mg AAE/g compared with oven-dried (63.30 ± 0.91 mg AAE/g) and convective air-dried (62.39 ± 0.11 mg AAE/g) samples. Overall, dried leaves exhibited a higher TAC when compared with fresh leaves. This observation was further noticed in TPC, DPPH radical scavenging activity, and TFC. Based on Figure 2, the total polyphenol content (TPC) in sun-dried samples (133.93 ± 3.76 mg GAE/g) was significantly higher (*p* < 0.05) compared with oven-dried, convective air-dried, and fresh leaf samples. This TPC value obtained was comparable to the TPC of *P. betle* leaves analysed by Dwijayanti et al. [8] at 128.92 ± 1.2 mg GAE/g and Vikrama et al. [18] at 130.00 ± 1.15 mg GAE/g. In addition to that, no significant difference (*p* > 0.05) was observed between oven-dried (122.69 ± 2.04 mg GAE/g) and convective air-dried (121.43 ± 2.64 mg GAE/g) samples. Figure 2 shows that the % inhibition in fresh *P. betel* leaves was the lowest, at 39.68 ± 0.10%, while sun-dried samples exhibited the highest % inhibition at 56.48 ± 0.11%. No significant difference (*p* > 0.05) was observed between oven-dried and convective air-dried samples with inhibition of 46.08 ± 0.48% and 42.46 ± 3.22%. Furthermore, the total flavonoid content (TFC) of *P. betle* ranged from 10.42 ± 0.25 to 81.25 ± 3.26 mg CE/g. Sun-dried samples had significantly higher (*p* < 0.05) TFC (81.25 ± 3.26 mg CE/g) compared with oven-dried and convective air-dried leaves at 68.19 ± 0.07 and 64.10 ± 0.16 mg CE/g respectively. As for alkaloid content, Figure 2 shows that the oven-dried samples possess the highest alkaloid content at 46.872 ± 0.153 mg/gm. No significant difference (*p* < 0.05) was observed between fresh and convective air-dried samples, while sun-dried had the lowest alkaloid content (45.684 ± 0.265 mg/gm) among the treatments. Alkaloids are widely found in plants and have various biological effects where they act primarily as defence components against pests or herbivores.

**Figure 3:**
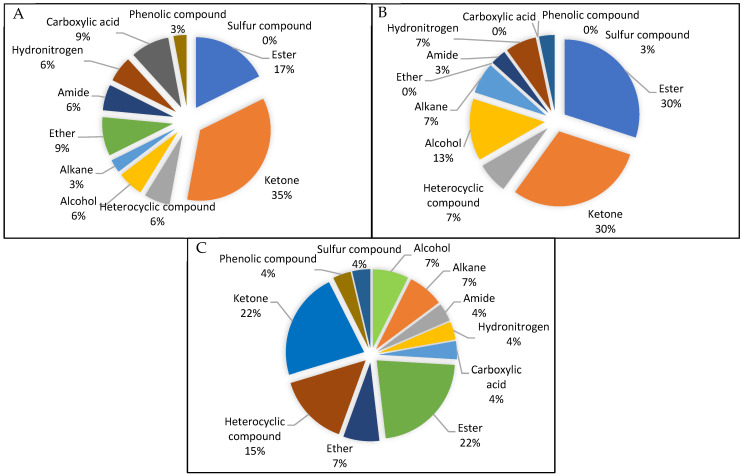
Pie diagram showing the percentage of phytochemical groups identified in *Piper betle* leaves for sun-dried samples (**A**), fresh samples (**B**), and the combined total (**C**).

The phytocompounds and their compound nature in sun-dried and fresh samples extracted using water are presented in Table 3. A total of 27 types of compounds were identified using GC-MS and were classified into esters (6), ketones (6), heterocyclic compounds (4), alcohols (2), alkanes (2), ethers (2), amides (1), amines (1), carboxylic acids (1), phenolic compounds (1), and sulfur compounds (1). The percentages of each compound in the sun-dried and fresh leaves and the total from both are presented in a pie chart (Figure 3).

## 3. Discussion

The decreased L* values in the dried leaves indicate that they became darker since L* values range from black at 0 to white at 100. However, the decrease was not significant (*p* > 0.05). On the other hand, the dried leaves also experienced an increase in a* and a decrease in b* values, indicating a loss of greenness as the a* measures red when positive and green when negative while b* measures yellow when positive and blue when negative. The natural greenness in leaves is associated with chlorophylls, but drying could lead to a loss of magnesium ions, which causes chlorophylls to be converted to pheophytins [19,20]. Higher L* values and lower a*/b* values are desirable in dried material [21]. Based on these criteria, both fresh and dried *P. betel* leaves presented desirable colour since there was no significant difference in the L* and a*/b* values between them.

From this experiment, dried *P. betle* leaves were found to contain higher amounts of antioxidants such as polyphenolics and higher antioxidant activity compared with fresh leaves, and this finding was consistent with a study by Thi et al. [14]. The dried samples had a higher TAC probably due to the drying treatment, which possibly induced structural changes in the leaf, thus increasing the extraction of antioxidant compounds by enhanced solvent and mass transfer [22]. Plant phenolics have garnered interest due to their effectiveness as free radical scavengers and antioxidants defending against ultraviolet radiation or pathogens [1,23,24]. The low TPC in fresh leaf samples was probably caused by the presence of an active enzyme called polyphenol oxidase that degrades the phenolic compounds, and in the dried samples, low water activity might have led to the inactivation of these enzymes resulting in higher levels of phenolic compounds in the samples [25]. Reports in the literature about the effects of drying on the TPC of plant samples vary. Some suggest that drying aids in breaking down the cell wall of plant materials, which helps release phenolics into the extracting solvent; conversely, drying can change the chemical structures of the phenolic compounds and cause them to adhere to other cellular components, which makes their extraction difficult [26]. Flavonoids and their derivatives are excellent free radical scavengers. Sun-dried samples had the highest TFC in the *P. betle* leaves. This could be due to the temperature in sun-dried samples being lower (38–45 °C) since the loss of flavonoids generally occurs at higher temperatures due to thermal degradation [27]. This might have led to a significant (*p* < 0.05) decrease in TFC value in oven-dried and convective air-dried samples. The drying time of sun-dried samples was also the longest, indicating a slower water loss rate, which might cause phenolics like flavonoids to increase as suggested by Zhang et al. [28].

DPPH is a stable free radical when interaction with an antioxidant receives electron or hydrogen atoms to neutralise its free radical character [29]. The DPPH free radical scavenging assay allows a preliminary assessment of a compound or sample of interest [9]. Based on our results, the sun-dried samples that had a significantly higher (*p* < 0.05) TPC and TFC also had a significantly higher (*p* < 0.05) DPPH radical scavenging activity. This trend was also followed at the opposite end of the spectrum, i.e., the fresh leaves had a significantly lower (*p* < 0.05) TPC and TFC, which corresponded to a significantly lower (*p* < 0.05) DPPH radical scavenging activity. This indicates that the drying influence of TPC and TFC of the leaf samples affected their antioxidant activity. Lou et al. [30] suggested that high TPC might contribute to the high antioxidant activity, which is probably due to the high hydrogen-donating ability of phenolic compounds [31]. The antioxidant activity of a leaf sample is also strongly related to its flavonoid content where the higher the content, the higher the radical scavenging activity [1,32,33]. Future studies can explore this further by testing multiple concentrations of the sun-dried leaf extract to optimise its antioxidant content and DPPH radical scavenging activity.

Based on the results, the major constituents found in the *P. betle* leaf extracts were esters (2%), ketones (22%), heterocyclic compounds (15%), alcohols (7%), alkanes (7%), and ethers (7%). The were differences in the type of compounds observed in the dried and fresh leaves upon extraction. For instance, an absence of phenolic type and carboxylic acid compounds was noticed in the aqueous extracts of the fresh leaves compared with the dried leaves. This could be due to their low amount in the fresh samples, coupled with the fact that carboxylic acids are usually extracted from aqueous solutions using organic solvents by the principle of reactive extraction [34]. In the dried samples, the drying process could have helped concentrate these compounds; therefore, their presence was more prominent. Conversely, there was almost double the percentage of alcohol- and ester-type compounds in the fresh leaf extracts compared with dried extracts. This could be due to the low boiling point of alcohols and esters, which have high heating sensitivity [35]. Furthermore, this was also consistent with a report by Zhang et al. [36] who observed that drying reduces ester content as they are hydrolysed during heat treatments.

Hydrazine, 1,2-dimethyl-; Ethyl aminomethylformimidate; glycerin; propanoic acid, 2-hydroxy-, methyl ester, (+/−)-; and 1,2-Cyclopentanedione were noticed in all of the sample extracts. Hydrazine, 1,2-dimethyl- is the only hydronitrogen compound found in our sample extracts. It has a molecular formula of C_2_H_8_N_2_ and a molecular weight of 60.1 g/mol. Hydrazine and its derivatives have been utilised to prevent the corrosion of boiler plants [37] and are widely utilised in the production of polymers, pharmaceuticals, and agricultural pesticides [38,39]. It is worth noting that daminozide, a type of plant growth regulator, can degrade to form dimethylhydrazine [40]. Its presence in our results could shed light on potential contaminants and safety risks posed by small-time vendors’ products since the leaf samples were obtained from a market. Pesticide residue has been reported in *P. betle* leaves recently [41]. Further studies are, therefore, recommended to explore this possibility in the samples obtained. Glycerin is also known as glycerol and has multiple uses across industries like pharmaceuticals and food [42]. To the best of our knowledge, this paper is the first to report on the presence of glycerin in the aqueous extract of the *P. betle* leaves. It is an alcoholic compound with a molecular weight of 92.09 g/mol and a molecular formula C_3_H_8_O_3_. It has been used as an alternative energy source for animal feeding. It possesses antioxidant properties as shown in a study by Araújo et al. [43] who demonstrated that supplementation with glycerine in diets of broilers increased its expression of uncoupling protein (UCP), which is related to mitochondrial function and glutathione peroxidase (GPx), which combats reactive oxygen species. Glycerin also has antimicrobial and anti-inflammatory properties [44]. Glycerin and Alpha-monopropionin are alcohol-related compounds found in our sample extracts. Ethyl aminomethylformimidate is an ester compound with 102.14 g/mol molecular weight and a molecular formula of C_4_H_10_N_2_O. Other ester compounds detected in the sample extracts were 2-Propenoic acid, methyl ester; acetic acid, hydroxy-, methyl ester; ethyl aminomethylformimidate; propanoic acid, 1-methylpropyl ester; propanoic acid, 2-hydroxy-, methyl ester, (+/−)-; and trimethylsilyl ethaneperoxoate. Oxirane, [(2-propenyloxy)methyl]- and silane, dimethyldi(but-3-enyloxy)- were ether-related products found in the extracts. 1,2-Cyclopentanedione is a ketone-based compound with a molecular weight of 98.1 g/mol and a molecular formula of C_5_H_6_O_2_. Other ketone compounds found in the extracts were acetoin; 1,2-Cyclopentanedione; 1,2-Cyclopentanedione, 3-methyl-; 2-Cyclopenten-1-one; 2-Cyclopenten-1-one, 2-hydroxy-3-methyl-; and 2-Cyclopenten-1-one, 3-ethyl-2-hydroxy-. 1,2-Cyclopentanedione, 3-methyl- has been shown to have anti-inflammatory properties via the suppression of pro-inflammatory gene expression through NF-κB signalling pathway modulation [45]. It has also been shown to possess radical scavenging properties by decreasing ONOO−, which is a by-product of reactive oxygen and nitrogen species that causes tissue damage via the formation of nitrotyrosine adducts glutathione (GSH) reductase [46]. We have also identified alkanes such as decane and octane, 4-ethyl-. Caprolactam and benzoic acid, 2,5-dimethyl- were amide and carboxylic acid compounds, respectively. Caprolactam is, more specifically, a cyclic amide with a molecular weight of 113.16 g/mol and a molecular formula of C_6_H_11_NO. Its derivatives are utilised in biomedical fields for drug delivery systems [47]. 2-Methoxy-4-vinylphenol is a phenolic compound while allyl mercaptan is a sulfur-based compound found in the extracts. 2-Methoxy-4-vinylphenol has a molecular weight of 150.17 g/mol and a molecular formula of C_9_H_10_O_2_. It is also a well-known styrene metabolite [48,49]. A study by Jeong et al. [50] showed that it possesses potent anti-inflammatory properties by inhibiting nitric oxide (NO), prostaglandins (PGE2), inducible NO synthase (iNOS), and cyclooxygenase-2 (COX-2) in cells. Several heterocyclic compounds were also identified in the sample extracts, such as 1,3,5,7-Cyclooctatetraene; benzofuran, 2,3-dihydro-; pyrrole; and styrene. While styrene is primarily a synthetic compound that can raise concerns about potential contaminants/ micropollutants, it has been identified in various natural plants, for example, in cinnamon by Fragnière et al. [51]. The authors acknowledged that incidences of styrene in food may not be related to exogenous contamination since it occurs naturally in foods. Overall, the bioactive compounds identified in the *P. betle* aqueous extracts possess medical properties such as antioxidant and anti-inflammatory activities that could have contributed to its role as a health-promoting agent and its role in the treatment of various ailments as claimed by traditional health practitioners.

## 4. Materials and Methods

### 4.1. Sample Preparation

*Piper betle* leaves were purchased from a local market in Petaling Jaya, Malaysia located approximately 6 km away from the Postharvest Laboratory, University of Malaya, Malaysia. They were immediately transported early in the morning (7 am MYT) in plastic bags and processed in the lab.

### 4.2. Drying Methods

Leaves were weighed, and 50 g was used for each drying experiment: oven-dried (55 °C), convective air-dried (60 °C), sun-dried (38–45 °C), and fresh leaves. The temperatures of the drying systems were measured using a thermometer. For sun-drying, the experiment was conducted from 10 am to 4 pm (sunlight directly reached the leaf samples in a tray without obstruction). Convective air-drying was conducted using a prototype self-built convective air-dryer [52]. The experiment was conducted in triplicates. The samples were spread evenly on the drying trays and left to dry until the sample weight was consistent for three readings. The dried leaves were pounded into powder under liquid nitrogen using a pestle and mortar.

### 4.3. Colour Measurement

Leaf colour was measured before and after drying with fresh leaves serving as a control. A chroma meter (Minolta CR-20, Tokyo, Japan) was used to measure the L*, a*, and b* values of fresh and dried *P. betle* leaves. The chroma meter was standardised with a white standard plate before three random measurements were taken for each sample. The values were recorded as L*, a* and b* and a*/b* based on Ali et al. [53] where L* measures the whiteness, with ranges from (black at 0 to white at 100), a* measures red when positive and green when negative, while b* measures yellow when positive and blue when negative.

### 4.4. Antioxidant Analysis

#### 4.4.1. Preparation of Extract

Leaves were prepared based on a method by Uribe et al. [54]. Briefly, a solid/liquid mixture with the ratio of 1:4 comprising a powdered sample and 80% methanol was prepared. The mixture with 80% methanol was chosen based on Jaiswal et al. [55]. The mixture was placed on an orbital shaker (Shellab Orbital Shaking Incubator S14, Cornelius, OR, USA) for 30 min at 200 rpm (room temperature). Next, the mixture was centrifuged at 6500 rpm for 10 min at 4 °C, and the resulting supernatant was used for subsequent analysis.

#### 4.4.2. Total Polyphenol Content

The samples’ total polyphenol content (TPC) was determined using Folin–Ciocalteu assay modified to a microscale [56]. A total of 0.79 mL of distilled water was added to 0.01 mL of the sample (gallic acid solution of known concentrations replaced the sample for the standard curve), followed by 0.05 mL of Folin–Ciocalteu reagent. After 1 min, 0.15 mL of sodium carbonate was added, and the mixture was left to stand at 25 °C for 2 h. The absorbance was measured at a wavelength of 750 nm using a spectrophotometer (Shimadzu UV-200-RS, MRC, Petah-Tikva, Israel). The equation from the gallic acid standard curve was y = 0.0056x, R2 = 0.9955, and results were expressed as mg of gallic acid equivalent (GAE) per gram of sample.

#### 4.4.3. Total Antioxidant Capacity

Total antioxidant capacity (TAC) was determined by the phosphomolybdenum method [57]. First, 1 mL of solution (0.6 M sulphuric acid, 4 mM ammonium molybdate, 28 mM sodium phosphate) of equal volumes was prepared. Subsequently, 0.01 mL of sample was added to the reagent mixture (80% methanol was used to replace the sample for blank), and the tubes were incubated for 90 min at 95 °C. The absorbance was read at 695 nm against blank once the sample cooled to room temperature. The equation of the ascorbic acid standard curve was y = 0.0018x, R2 = 0.9981. The results were expressed as milligrams of ascorbic acid equivalent (AAE) per gram of plant material.

#### 4.4.4. DPPH Radical Scavenging Assay

The DPPH assay was carried out via a method described by Bae and Suh [54]. 0.1 mM DPPH solution was prepared with 80% methanol. A total of 1 mL of the solution was added to 500 µL of samples. The radical scavenging activity was calculated using the formula:

% DPPH inhibition = (Acontrol − Asample/Acontrol) × 100

where Acontrol: absorbance of control; Asample: absorbance of the sample.

The results were reported as % inhibition.

#### 4.4.5. Total Flavonoid Content

Total flavonoid content (TFC) was determined using the colourimetric method based on Sakanaka et al. [58]. Briefly, 1.25 mL of distilled water was added to the 0.25 mL sample. The sample was replaced with (+)-standard catechin solution for standard curve construction. After that, 75 μL of a 5% sodium nitrite solution was added and left at room temperature for 6 min. Then, 150 μL of a 10% aluminium chloride solution was added, and the mixture was incubated for 5 min; then, 0.5 mL of 1 M sodium hydroxide was added. Distilled water was used to bring the mixture up to 2.5 mL. The absorbance was measured at 510 nm. The catechin standard curve had an equation of y = 0.0135x, R2 = 0.9943, and the results obtained were reported as milligrams of catechin equivalent (CE) per gram of sample.

### 4.5. Gas Chromatography-Mass Spectroscopy (GCMS) Analysis

Various households tend to prepare *P. betle* leaf extracts using water for consumption. Therefore, based on the antioxidant results, we extended our study by comparing phytocompounds found in aqueous extracts of sun-dried and fresh leaves using GCMS.

#### 4.5.1. Sample Preparation

*P. betle* leaves were purchased from a local market in Petaling Jaya, Malaysia, located approximately 6 km away from the Postharvest Laboratory, University of Malaya, Malaysia. Leaves (10 g) were subjected to (a) the drying treatment under the sun and (b) no treatment (fresh) for GC-MS analysis. The leaves were ground into powder under liquid nitrogen using a pestle and mortar. Then, the powdered samples were extracted in aqueous solutions via 2 different methods. The first method was based on Madi et al. [59] with slight modifications where powdered leaves were soaked in hot distilled water (100 °C) in a 1:10 solid/liquid ratio for 30 min. Once the suspension settled at room temperature, it was filtered using Whatman No. 1 filter paper. The second method involved soaking the leaves in distilled water (1:10 solid/liquid ratio) and leaving them overnight. The following day, the extract was filtered using Whatman No. 1 filter paper. Filtered extracts from both the first and second extraction methods were then concentrated in a rotary evaporator and resuspended at 1 mg/mL.

#### 4.5.2. Screening of Compounds

The characterisation of the phytochemicals in the leaves was performed using GC-MS QP2010 Plus (Shimadzu, Tokyo, Japan). GC was conducted in the temperature programming mode with an Rtx-5MS column (0.25 mm, 30 m). The initial column temperature was 40 °C for 1 min. The injection temperature was 300 °C (splitless mode); the oven temperature was programmed from 40 °C and held for 5 min to 160 °C at a rate of 4 °C/min, then to 280 °C at a rate of 5 °C/min and held for 15 min. Identifications were based on mass spectral matching with standard compounds in the NIST library with a similarity index of at least 80% [33]. The relative amounts of individual components were expressed as percent peak areas relative to the total peak area.

### 4.6. Statistical Analysis

Results were expressed as mean ± SD from three experiments (*n* = 3). One-way ANOVA was performed using the Statistical Package for the Social Sciences (SPSS) v23.0 (IBM, Armonk, NY, USA) for each treatment. If significance was shown (*p* < 0.05), a post hoc analysis, (Tukey HSD test) was performed to identify which pair(s) in each column was/were statistically different. The same letter denotes mean values that are not significantly different (*p* > 0.05).

## 5. Conclusions

Sun-drying has often been employed by the masses to dry products because it is easily accessible, requires low skill, and is low cost. This is crucial to those who rely on small-scale plantations of this crop like home-grown or small farms as a reliable source of income. However, it would be disadvantageous if the quality of the leaves is not proportionate to the work that was invested, especially for a health-conscious consumer market. To this end, many studies have shown varying reports on sun-drying’s efficacy for leaf products, owing to the possible thermal and UV-induced degradation of bioactive compounds in them. Therefore, our study provided important evidence in support of the use of sun-drying as a preparation method for *P. betle* leaves. This is because the sun-dried samples showed a significantly higher (*p* < 0.05) TAC, TFC, TPC, and alkaloid content, and DPPH radical scavenging activity compared with all other drying treatments and fresh samples. This is important for locals who wish to market sun-dried *P. betle* leaves as a high-antioxidant natural product. Furthermore, the aqueous extracts revealed important bioactive compounds like 1,2-Cyclopentanedione, 3-methyl-, glycerin, and 2-Methoxy-4-vinylphenol that have strong antioxidant and anti-inflammatory properties, while the others have important applications in various fields ranging from pharmaceuticals to the food industry. Future studies are recommended to assess the individual composition of each leaf sample and evaluate the microbial and pesticide residue to provide a more comprehensive understanding of the safety of these leaves for consumption.

## Figures and Tables

**Table 1:** Taxonomic classification of *Piper betle*.

Level	Classification
Kingdom	Plantae
Division	Magnoliophyta
Class	Magnoliopsida
Order	Piperales
Family	Piperaceae
Genus	*Piper*
Species	*betle*

**Table 2:** Colour of *Piper betle* leaves subject to various drying methods.

	Drying Method	Fresh	Oven	Convective	Sun
Colour Parameter					
L*		41.8 ± 4.53 ^a^	33.17 ± 2.96 ^a^	33.13 ± 2.91 ^a^	33.47 ± 2.29 ^a^
a*		−7.53 ± 0.54 ^a^	−5.13 ± 0.86 ^b^	−3.20 ± 0.67 ^bc^	−2.70 ± 0.67 ^c^
b*		28.07 ± 6.76 ^a^	16.70 ± 2.07 ^ab^	15.97 ± 2.38 ^ab^	15.23 ± 1.53 ^b^
a*/b*		−0.29 ± 0.10 ^a^	−0.31 ± 0.03 ^a^	−0.21 ± 0.07 ^a^	−0.18 ± 0.05 ^a^

Results were expressed as mean ± SD from three experiments (*n* = 3). 1 One-way ANOVA was carried out for each treatment (*p* < 0.05) and showed significance. Post hoc analysis, (Tukey HSD test) was used to identify which pair(s) in each column was/were statistically different. The same letter denotes mean values that are not significantly different (*p* > 0.05).

**Table 3:** List of phytocompounds identified by GC-MS in the aqueous extracts of *Piper betle* leaves using various drying and extraction methods.

**Drying Method: Sun-Dried** **Extraction Method: First**				
**Peak**	**R Time**	**Area %**	**Name**	**Nature of Compound**
1	3.144	43.56	Hydrazine, 1,2-dimethyl-	Hydronitrogen
2	3.262	4.04	Ethyl aminomethylformimidate	Ester
3	3.598	1.55	Acetic acid, hydroxy-, methyl ester	Ester
4	3.701	1.49	Acetoin	Ketone
5	3.974	10.35	Glycerin	Alcohol
6	4.119	2.30	Propanoic acid, 2-hydroxy-, methyl ester, (+/−)-	Ester
7	6.297	3.34	2-Cyclopenten-1-one	Ketone
8	8.110	0.87	1,3,5,7-Cyclooctatetraene	Heterocyclic compound
9	9.832	15.50	1,2-Cyclopentanedione	Ketone
10	12.656	2.21	Oxirane, [(2-propenyloxy)methyl]-	Ether
11	13.926	1.21	1,2-Cyclopentanedione, 3-methyl-	Ketone
12	14.086	2.20	1,2-Cyclopentanedione, 3-methyl-	Ketone
13	17.801	0.88	2-Cyclopenten-1-one, 3-ethyl-2-hydroxy-	Ketone
14	19.695	1.15	Silane, dimethyldi(but-3-enyloxy)-	Ether
15	21.990	0.86	Benzofuran, 2,3-dihydro-	Heterocyclic compound
16	23.041	1.88	Caprolactam	Amide
17	30.767	6.62	Benzoic acid, 2,5-dimethyl-	Carboxylic acid
**Drying Method: Sun-Dried** **Extraction Method: Second**				
**Peak**	**R time**	**Area %**	**Name**	**Nature of compound**
1	3.133	45.09	Hydrazine, 1,2-dimethyl-	Hydronitrogen
2	3.258	3.78	Ethyl aminomethylformimidate	Ester
3	3.599	1.46	Acetic acid, hydroxy-, methyl ester	Ester
4	3.699	1.56	Acetoin	Ketone
5	3.972	9.95	Glycerin	Alcohol
6	4.121	1.38	Propanoic acid, 2-hydroxy-, methyl ester, (+/−)-	Ester
7	6.297	2.77	2-Cyclopenten-1-one	Ketone
8	9.819	14.12	1,2-Cyclopentanedione	Ketone
9	12.651	1.88	Octane, 4-ethyl-	Alkane
10	13.921	1.19	1,2-Cyclopentanedione, 3-methyl-	Ketone
11	14.083	2.03	1,2-Cyclopentanedione, 3-methyl-	Ketone
12	17.799	1.09	2-Cyclopenten-1-one, 3-ethyl-2-hydroxy-	Ketone
13	19.693	1.04	Silane, dimethyldi(but-3-enyloxy)-	Ether
14	23.036	2.04	Caprolactam	Amide
15	25.080	0.54	2-Methoxy-4-vinylphenol	Phenolic compound
16	30.760	10.09	Benzoic acid, 2,5-dimethyl-	Carboxylic acid
**Drying Method: None (Fresh Leaves)** **Extraction Method: First**				
**Peak**	**R time**	**Area %**	**Name**	**Nature of compound**
1	3.031	16.59	Hydrazine, 1,2-dimethyl-	Hydronitrogen
2	3.253	9.74	Ethyl aminomethylformimidate	Ester
3	3.344	2.41	Trimethylsilyl ethaneperoxoate	Ester
4	3.985	29.41	Glycerin	Alcohol
5	4.133	3.87	Propanoic acid, 2-hydroxy-, methyl ester, (+/−)-	Ester
6	4.366	2.45	2-Propenoic acid, methyl ester	Ester
7	4.653	3.56	Alpha-monopropionin	Alcohol
8	6.341	4.63	2-Cyclopenten-1-one	Ketone
9	9.708	10.65	1,2-Cyclopentanedione	Ketone
10	9.804	5.77	1,2-Cyclopentanedione	Ketone
11	12.666	2.42	Decane	Alkane
12	14.070	6.26	2-Cyclopenten-1-one, 2-hydroxy-3-methyl-	Ketone
13	22.891	2.23	Caprolactam	Amide
**Drying Method: None (Fresh Leaves)** **Extraction Method: Second**				
**Peak**	**R time**	**Area %**	**Name**	**Nature of compound**
1	3.041	19.88	Hydrazine, 1,2-dimethyl-	Hydronitrogen
2	3.200	1.15	Ethyl aminomethylformimidate	Ester
3	3.261	9.83	Allyl mercaptan	Sulfur compound
4	3.342	2.20	Trimethylsilyl ethaneperoxoate	Ester
5	3.606	1.54	Acetic acid, hydroxy-, methyl ester	Ester
6	3.982	27.88	Glycerin	Alcohol
7	4.129	3.60	Propanoic acid, 2-hydroxy-, methyl ester, (+/−)-	Ester
8	4.427	2.08	Pyrrole	Heterocyclic compound
9	4.554	1.40	Glycerin	Alcohol
10	4.646	1.82	Propanoic acid, 1-methylpropyl ester	Ester
11	6.311	4.64	2-Cyclopenten-1-one	Ketone
12	8.115	0.97	Styrene	Heterocyclic compound
13	9.714	11.16	1,2-Cyclopentanedione	Ketone
14	9.792	4.65	1,2-Cyclopentanedione	Ketone
15	12.655	3.48	Decane	Alkane
16	13.922	1.70	1,2-Cyclopentanedione, 3-methyl-	Ketone
17	14.061	2.03	1,2-Cyclopentanedione, 3-methyl-	Ketone

R time = Retention time.

## Data Availability

The data presented in this study are available in article.
